# Powerful Inference with the D-Statistic on Low-Coverage Whole-Genome Data

**DOI:** 10.1534/g3.117.300192

**Published:** 2017-12-01

**Authors:** Samuele Soraggi, Carsten Wiuf, Anders Albrechtsen

**Affiliations:** * Department of Mathematical Sciences, Faculty of Science, University of Copenhagen, 2100, Denmark; † Center for Bioinformatics, Faculty of Science, University of Copenhagen, 2100, Denmark

**Keywords:** admixture, gene flow, introgression, D-statistic, ABBA–BABA test, tree test, four-population test, ANGSD, next-generation sequencing data, low depth

## Abstract

The detection of ancient gene flow between human populations is an important issue in population genetics. A common tool for detecting ancient admixture events is the D-statistic. The D-statistic is based on the hypothesis of a genetic relationship that involves four populations, whose correctness is assessed by evaluating specific coincidences of alleles between the groups. When working with high-throughput sequencing data, calling genotypes accurately is not always possible; therefore, the D-statistic currently samples a single base from the reads of one individual per population. This implies ignoring much of the information in the data, an issue especially striking in the case of ancient genomes. We provide a significant improvement to overcome the problems of the D-statistic by considering all reads from multiple individuals in each population. We also apply type-specific error correction to combat the problems of sequencing errors, and show a way to correct for introgression from an external population that is not part of the supposed genetic relationship, and how this leads to an estimate of the admixture rate. We prove that the D-statistic is approximated by a standard normal distribution. Furthermore, we show that our method outperforms the traditional D-statistic in detecting admixtures. The power gain is most pronounced for low and medium sequencing depth (1–10×), and performances are as good as with perfectly called genotypes at a sequencing depth of 2×. We show the reliability of error correction in scenarios with simulated errors and ancient data, and correct for introgression in known scenarios to estimate the admixture rates.

An important part of the understanding of a population’s history and its genetic variability is past contacts with other populations. Such contacts could result in gene flow and admixture between populations and leave traces of a population’s history in genomic data. In fact, the study of gene flow between populations has been used to uncover demographic histories of many species, including human and archaic human populations (Patterson *et al.* 2012; Raghavan *et al.* 2013, 2015; Green *et al.* 2010; Reich *et al.* 2009, 2010, 2011; Wall *et al.* 2013; Rasmussen *et al.* 2010, 2014; Lalueza-Fox and Gilbert 2011; Skoglund *et al.* 2015).

The study of the history of human populations using both modern and ancient human genomes has become increasingly topical with the recent availability of new high-throughput sequencing technologies (Stoneking and Krause 2011) such as next-generation sequencing (NGS) (Black *et al.* 2015). These technologies have made it possible to obtain massive quantities of sequenced DNA data even from ancient individuals, including an Anzick Clovis individual from the late Pleistocene (Rasmussen *et al.* 2014), a Neandertal individual (Green *et al.* 2010), and a Paleoamerican individual (Chatters 2000).

There are many different methods for inferring and analyzing admixture events using genome-scale data. Popular methods such as STRUCTURE (Pritchard *et al.* 2000) and ADMIXTURE (Alexander *et al.* 2009) estimate how much a sampled individual belongs to *K* clusters, which can often be interpreted as the individual’s admixture proportional to the *K* populations. However, these approaches are not appropriate for detecting ancient gene flow and do not work well with a limited number of individuals per population.

A recent alternative to the above methods is the D-statistic. The D-statistic is based on the di-allelic patterns of alleles between four groups of individuals, and provides a way to test the correctness of a hypothetical genetic relationship between the four groups (see Figure 1). A variant of the D-statistic (called the *F*_4_-statistic) was first used in Reich *et al.* (2009) to show that subgroups of the Indian Cline group are related to external populations in terms of gene flow. The amount of gene flow can also be estimated using the *F*_4_-statistic (Wall *et al.* 2013).

**Figure 1:**
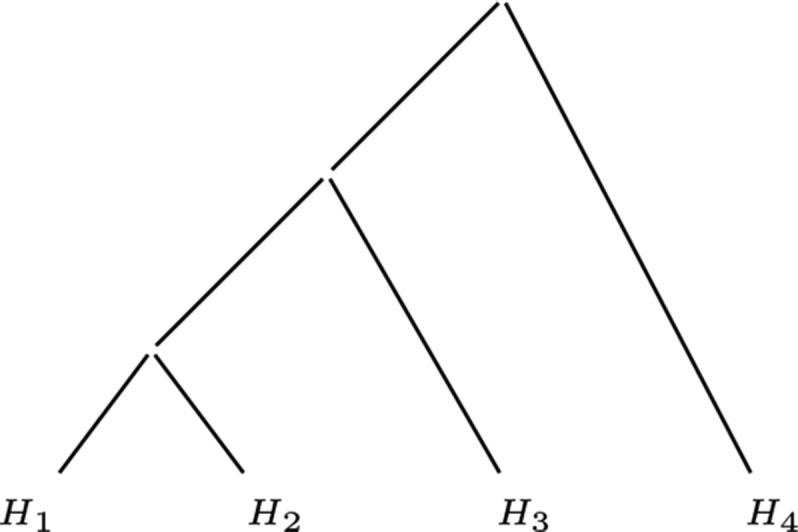
Tree topology for the D-statistic. Hypothesis of genetic relationship between four populations, $H_{1},H_{2},H_{3},H_{4}.$

In a pivotal study (Green *et al.* 2010), the D-statistic was used to show that three non-African individuals were more genetically similar to the Neandertal sequence than African San and Yoruban individuals were. Moreover, it has been shown that the East Asian populations have greater amounts of Neandertal shared genetic material (Wall *et al.* 2013).

Based on use of the D-statistic on many Old World and Native American individuals, gene flow into some Native American populations has been suggested, for instance, evidence of admixture from Australasian populations into New World populations (Raghavan *et al.* 2015; Skoglund *et al.* 2015).

In another study, the affinity between the Anzick genome and the Native American genome was analyzed with the D-statistic to compare different hypotheses regarding their ancestry (Rasmussen *et al.* 2014). Using the D-statistic, it has been reported based on the remains of an individual from the Mal’ta population in south-central Siberia, that this population contributed to the gene pool of modern-day Native Americans, with no close affinity to East Asians (Raghavan *et al.* 2013).

The first use of the D-statistic was based on a sampling approach that allowed the test to be performed without the need to call single-nucleotide polymorphisms (SNPs) or genotypes (Green *et al.* 2010). This approach is still widely used, and the available computational tools implementing it include the doAbbababa program ANGSD (Analysis of Next Generation Sequencing Data) (Nielsen *et al.* 2011) (supporting low-depth NGS data) and the fourpop program of TreeMix (Pickrell and Pritchard 2012) (supporting di-allelic genotype data and microsatellite data). The program qpDstat of ADMIXTOOLS (Patterson *et al.* 2012) computes the D-statistic from populations with multiple individuals from di-allelic genotype data. The program doAbbababa relies on sampling one base from every locus, using the sequenced reads to define the sampling probabilities.

The D-statistic is often applied to scenarios involving ancient individuals, which are commonly affected by deamination, *i.e.*, the natural degradation of DNA after death of the organism, which leads to there being few molecules remaining in ancient specimens and often results in a low sequencing depth. Furthermore, deamination can cause high frequency of specific transitions of the bases, low quality of the SNPs, and very low depth of the data. The current methods for the D-statistic can be very ineffective and unreliable when applied to ancient data, since both sampling and genotype calling procedures are subject to high uncertainty.

The focus of this paper is to address the problems stated above. We propose a D-statistic—implemented in the program doAbbababa2 of ANGSD—that supports low-depth NGS data and is calculated using all reads of the genomes, and therefore allows for the use of >1 individual per group. We prove that the improved D-statistic is approximated by a standard normal distribution and, using both simulated and real data, we show how this approach greatly increases the sensitivity of gene-flow detection and thus improves the reliability of the method, in comparison with sampling a single read. We also illustrate that it is possible to correct for type-specific error rates in the data, so that the reads used to calculate the D-statistic will not bias the result owing to type-specific errors. Moreover, our improved D-statistic can remove the effects of known introgression from an external population into $H_{1},$ $H_{2}$, or $H_{3},$ and indirectly estimates the admixture rate.

## Materials and Methods

This section introduces the traditional D-statistic and the theory that leads to its approximation as a normal distribution. Thereafter, we explain how to extend the D-statistic to use multiple individuals per population, without genotype calling and while still preserving the same approximation property of the D-statistic. Last, we will show how to deal with type-specific errors and introgression from a population external to the tree topology.

### Standard D-statistic

The objective of the D-statistic is to assess whether the tree of Figure 1 that relates four present-day populations, $H_{1},H_{2},H_{3},H_{4},$ is correct. When $H_{4}$ is an outgroup, the correctness of the tree corresponds to the absence of gene flow between $H_{3}$ and either $H_{2}$ or $H_{1}.$ This objective is achieved by developing a statistical test based on the allele frequencies and a null hypothesis $H_{0}$ that the tree is correct and without gene flow. We limit the explanation to a di-allelic model with alleles A and B to keep the notation uncluttered; the extension to a four-allele model is fairly straightforward. We do not make an assumption on which allele is derived, but we assume that B is the nonoutgroup allele. Population $H_{4}$ is an outgroup that splits off from the other branches at the root of the tree. For each population $H_{j},\;j=1,2,3,4,$ in the tree, we consider the related allele frequencies $x_{j}.$

For each population $H_{j},$ the observed data consist of a certain number of individuals sequenced without error. At every locus *i* there are $n_{j}^{i}$ sequenced bases observed from aligned reads. We consider only the *M* loci for which there is at least one sequenced base from aligned reads in all four groups. Moreover, in this theoretical treatment, we allow the number *M* of loci to grow to infinity. Assume that at a locus *i* the allele frequencies in the four groups of individuals $x_{i}:=(x_{1}^{i},x_{2}^{i},x_{3}^{i},x_{4}^{i}),$ and let ${\hat{x}}_{i}:=({\hat{x}}_{1}^{i},{\hat{x}}_{2}^{i},{\hat{x}}_{3}^{i},{\hat{x}}_{4}^{i})$ be an unbiased estimator of $x_{i},$ such as the relative frequencies of the allele A in each population.

The D-statistic focuses on di-allelic sites where the differences are observed within the pairs $\left(H_{1},H_{2}\right)$ and $\left(H_{3},H_{4}\right).$ Consider a random allele drawn from each of the four groups of genomes and the resulting combination of the four alleles. We are interested in two patterns:

- ABBA, meaning that we have the same allele in populations $H_{1}$ and $H_{4}$, and another allele from the individuals in populations $H_{2}$ and $H_{3};$
- BABA, where one allele is shared by individuals in populations $H_{1}$ and $H_{3}$, and the other allele by individuals in populations $H_{2}$ and $H_{4}.$

The tree of Figure 1 is subject to independent genetic drifts of the allele frequencies along each of its branches. Consequently, the probabilities of ABBA and BABA patterns, which are conditional only on population frequencies, would rarely be the same. Therefore, it is interesting to focus on their expected values with respect to the frequency distribution:

$$P\left(ABBA_{i}\right)=E[x_{1}^{i}x_{4}^{i}\left(1-x_{2}^{i}\right)\left(1-x_{3}^{i}\right)+\left(1-x_{1}^{i}\right)\left(1-x_{4}^{i}\right)x_{2}^{i}x_{3}^{i}]$$ (1)

$$P\left(BABA_{i}\right)=E[\left(1-x_{1}^{i}\right)x_{2}^{i}\left(1-x_{3}^{i}\right)x_{4}^{i}+x_{1}^{i}\left(1-x_{2}^{i}\right)x_{3}^{i}\left(1-x_{4}^{i}\right)]$$ (2)

To verify that allele A is shared between genomes in $H_{1},H_{3}$ as often as it is shared between genomes in $H_{2},H_{3},$ we require as null hypothesis that at each *i*th locus the probability (1) equals the probability (2). This condition can be written as:

$$H_{0}:E\left[\left(x_{1}^{i}-x_{2}^{i}\right)\left(x_{3}^{i}-x_{4}^{i}\right)\right]=0,\;\text{for}\;i=1,\ldots ,M,$$

where the expectation is the difference between (1) and (2).

Using the empirical frequencies of the alleles as unbiased estimators for the population frequencies, we define the D-statistic as the following normalized test statistic:

$$D_{M}:=\frac{X_{\left(M\right)}}{Y_{\left(M\right)}}=\frac{\sum_{i=1}^{M}({\hat{x}}_{1}^{i}-{\hat{x}}_{2}^{i})({\hat{x}}_{3}^{i}-{\hat{x}}_{4}^{i})}{\sum_{i=1}^{M}\left({\hat{x}}_{1}^{i}+{\hat{x}}_{2}^{i}-2{\hat{x}}_{1}^{i}{\hat{x}}_{2}^{i}\right)({\hat{x}}_{3}^{i}+{\hat{x}}_{4}^{i}-2{\hat{x}}_{3}^{i}{\hat{x}}_{4}^{i})}\;$$ (3)

The values $X_{\left(M\right)}$ and $Y_{\left(M\right)}$ are the numerator and denominator, respectively. Using $Y_{\left(M\right)}$ to normalize the numerator leads to the interpretation of $D_{M}$, as the difference over all loci of the probabilities of having an ABBA or a BABA event, conditional on the assumption that only ABBA or BABA events are possible.

Appendix 1 shows that under the hypothesis $H_{0}$, the test statistic can be approximated by a standard normal variable. Specifically, the approximation holds with a proper rescaling, since $D_{M}$ would narrow the peak of the Gaussian around zero for large *M* (note that this rescaling is an embedded factor in the estimation of the variance of $D_{M}$ using the block jackknife method (Busing *et al.* 1999) in the software implementation of ANGSD). More generally, the treatment could be extended to blockwise independence of the allele counts to take into account linkage disequilibrium.

The convergence results of Appendix 1 apply to the following special cases of the D-statistic:

1. the original D-statistic $D_{M}$ calculated by sampling a single base from the available reads (Green *et al.* 2010) to estimate the sampling probabilities;
2. the D-statistic $D_{M}$ evaluated by substituting the frequencies ${\hat{x}}_{j}^{i}$ with the estimated population frequencies $\;{\hat{q}}_{j}^{i}$ defined in equation 4 for multiple individuals (see Appendix 2);
3. the D-statistic $D_{M}$ evaluated only over loci where the outgroup is mono-allelic, such as when the chimpanzee is set as an outgroup to test for gene flow from the Neandertal population into modern out-of-Africa populations (Green *et al.* 2010).

### Multiple individuals per group

The D-statistic defined in equation 3 is calculated using population frequencies. In the case where only one individual per population is chosen, it is easy to get an estimate of the populations’ frequencies by simply counting observed bases. In what follows, we are interested in getting a meaningful estimate of the frequencies in the case where we want to use all the available sequenced individuals without calling genotypes.

This is done using a weighted sum of the estimated allele frequencies for each individual in every group. Assume that given the allele frequency $x_{j}^{i},\;j=1,2,3,4,$ at locus *i* for the *j*th population, we model the observed data as independent binomial trials with parameters $n_{j}^{i}$ and $x_{j}^{i},$ where $n_{j}^{i}$ is the number of trials. We take the frequency of allele A in the reads of each *j*th population as an unbiased estimator of the population frequency. Let $N_{j}$ be the number of individuals in population *j*. For the first individual within the *j*th population, let $x_{j,l}^{i}$ be the frequency of allele A at locus *i*, with estimator ${\hat{x}}_{j,l}^{i}$ the frequency of allele A for $l=1,...,\;N_{j}$. Define ${\hat{q}}_{j}^{i}$ as the weighted sum

$${\hat{q}}_{j}^{i}:=\overset{N_{j}}{\underset{l=1}{\sum }}w_{j,l}^{i}\cdot \;{\hat{x}}_{j,l}^{i},$$ (4)

where each $w_{j,l}^{i}$ is a weight that is proportional to a quantity depending on $n_{j,l}^{i}$, the number of sequenced bases at locus *i* for individual *l*:

$$w_{j,l}^{i}\propto \frac{2n_{j,l}^{i}}{n_{j,l}^{i}+1}$$. (5)

The estimator ${\hat{q}}_{j}^{i}$ in equation (4) is an estimator for the *j*th population frequency at locus *i* with minimal variance (the derivation of the weights as minimizer of the frequency estimator’s variance can be found in Appendix 2). Substituting the estimated population frequencies in equation (3) with the weighted estimators determined by equation (4), it is possible to account for multiple individuals per population. Since the weighted estimator is also unbiased, it does not affect the approximation of the D-statistic with a standard normal distribution.

A first application of this method has been the estimation of population frequencies to reveal signatures of natural selection (Li *et al.* 2010). The weights have a strong impact on loci with a low number of reads, where they assume a low value, leading to a stronger impact of population frequency estimated from high-depth individuals in each group.

### Error estimation and correction

The study of genetic relationships between populations often involves the use of ancient genomes that are subject to high error rates. We introduce error correction following the idea illustrated in Orlando *et al.* (2013), to take errors into account and to obtain a more reliable D-statistic.

Estimation of type-specific error rates is possible using two individuals (one affected by type-specific errors and one sequenced without errors) and an outgroup, denoted by T, R, and O, respectively. Those individuals are considered in the tree ((T,R),O) (see Appendix 3).

After the error matrix is estimated for each individual, it is possible to obtain error-adjusted frequencies of alleles in locus *i* through the following matrix–vector product:

$$p_{G}^{i}=e^{-1}p_{T}^{i}.$$ (6)

where $p_{G}^{i}$ and $p_{T}^{i}$ are the true and observed vectors of allele frequencies locus *i*, respectively, and e is the 4 × 4 type-specific error matrix whose entry e(a,b) is the probability of observing a base of type *b* when the true base is of type *a*. Note that estimating e and correcting the allele frequencies is a process best applied before the calculation of weighted allele frequencies for multiple individuals.

Using error-corrected estimators of the population frequencies to calculate the D-statistic does not prevent it from being approximated by a standard normal distribution, because the error-corrected estimators are unbiased for the true population frequency (see Appendix 3).

According to equation (6), one is able to perform the error correction at every locus for every individual. In this way, it is possible to build a weighted frequency estimator for each population after the error correction. However, the implementation of equation (6) involves the inversion of a matrix and a matrix–vector multiplication at every locus for each individual in all populations. Moreover, as a consequence of the error estimation, there might be negative entries of the inverse $e^{-1}$, which might cause the product of equation (6) to result in negative entries in the vector $p_{G}^{i}.$

Consequently, we decided to implement a less precise version of the error correction that is applied to each whole group of individuals instead of every single individual. Assume that the populations’ frequencies have been estimated from equation (4), and that it is possible to estimate the probabilities of the 256 allele combinations AAAA, AAAC,…, TTTT between the four populations.

In each *j*th population of individuals, let $e_{\left(j\right)}$ be the mean of their error matrices. Then build the error matrix for the four groups, E. This has dimension 256 × 256, and its entry $\left(a_{1:4},b_{1:4}\right),$ where $a_{1:4}=\left(a_{1},a_{2},a_{3},a_{4}\right)$ and $b_{1:4}=\left(b_{1},b_{2},b_{3},b_{4}\right)$ are two possible allele patterns of the four populations, is defined as the probability of observing $b_{1:4}$ instead of $a_{1:4},$ assuming independence of the error rates between the four populations:

$$E\left(a_{1:4},b_{1:4}\right)=e_{1}\left(a_{1},b_{1}\right)\cdot e_{2}\left(a_{2},b_{2}\right)\cdot e_{3}\left(a_{3},b_{3}\right)\cdot e_{4}\left(a_{4},b_{4}\right).$$

The equation states that the change from pattern $a_{1:4}$ to $b_{1:4}$ happens with a probability that is the product of the error rates of each population. Note that each error rate is the sum of the error rates of each individual in that population, and so does not take into account how every individual is weighted according to the frequency estimator of equation (4).

Let ***P****_error_* be the vector of length 256 that contains the estimated probabilities of observing allele patterns between the four populations, affected by type-specific errors. Denote by ***P****_corr_* the vector containing the estimated probabilities of patterns not affected by errors. With an approach similar to the one leading to equation 6, it holds that

$$P_{corr}=E^{-1}P_{error}$$

Using the error-corrected estimated probabilities of combinations of alleles of the type ABBA and BABA, it is then possible to calculate the numerator and denominator of the D-statistic. This procedure is fast, but it has the drawback that in every group the error matrix takes into account every individual within a population without its associated weight from equation 5. This means that the portion of alleles related to individuals with lower weights might undergo an excessive error correction.

### Correction for introgression from an external population

The improved D-statistic proves to be very sensitive to introgression, but a hypothesized genetic relationship might be rejected because of an admixture involving a population not part of the considered tree. We propose a way to correct this issue and obtain an estimate of the amount of introgression when the source of gene flow is available.

In this section, we analyze the case in which the null hypothesis might be rejected in favor of the alternative hypothesis, but the cause of rejection is not the presence of gene flow between $H_{3}$ and either $H_{1}$ or $H_{2},$ but instead gene flow between an external population $H_{5}$ and either $H_{2}$ or $H_{1}.$ Consider the case of Supplemental Material, Figure S3A in File S1, where the null hypothesis might be rejected because of introgression from an external population $H_{5}$ into $H_{2}$ with rate α. We assume that the external sample for $H_{5}$ represents the population that is the source of introgression. Consider $H_{2}$ to be the population subject to introgression from $H_{5},$ and define $H_{2}^{\prime }$ as the same population when it has not undergone admixture.

The four-population subtrees of interest (see Figure S3 in File S1) are $T_{1:4}=\left(\left(\left(H_{1},H_{2}\right)H_{3}\right)H_{4}\right),$ which includes the four-population tree excluding the admixing population; $T_{out}=\left(\left(\left(H_{1},H_{5}\right)H_{3}\right)H_{4}\right),$ where the population source of introgression replaces the admixed population; and $T_{un}=\left(H_{1}\left(H_{2}^{\prime }\left(H_{3},H_{4}\right)\right)\right),$ in which $H_{2}$ has not yet undergone admixture and therefore reflects the null hypothesis *H*_0_.

Consider the patterns of four alleles for the three subtrees mentioned above, whose estimated probabilities are respectively denoted as $p_{1:4},$ $p_{out}$, and $p_{un}.$ Using the frequency estimators of equation (4), it is possible to estimate $p_{1:4}$ and $p_{out},$ but not $p_{un}$ since $H_{2}^{\prime }$ is not an observed population.

Assume that testing with the D-statistic on the tree $T_{1:4}$ leads to a rejection of $H_{0}$ because the allele frequencies of $H_{2}$ are altered by the gene flow from $H_{5}.$ In fact, any combination of four alleles observed in $T_{1:4}$ has probability

$$p_{1:4}=\left(1-\alpha \right)p_{un}+\alpha p_{out}.$$

By solving for $p_{un}$ it follows that

$$p_{un}=\frac{1}{1-\alpha }\left(p_{1:4}-\alpha p_{out}\right).$$ (7)

Note that if the admixture proportion α is known, then admixture correction is possible. If α is not known and we assume the tree is accepted for E [*D_un_*] = 0, where $D_{un}$ is the D-statistic related to the tree $T_{un},$ then α can be estimated. In this case, $p_{un}$ has to be determined for all values of α, and the correct value will be the one for which E [*D_un_*] = 0. In this way, an estimate of the admixture rate was obtained for the topology of Figure S3A in File S1.

### Simulations

**Figure 2:**
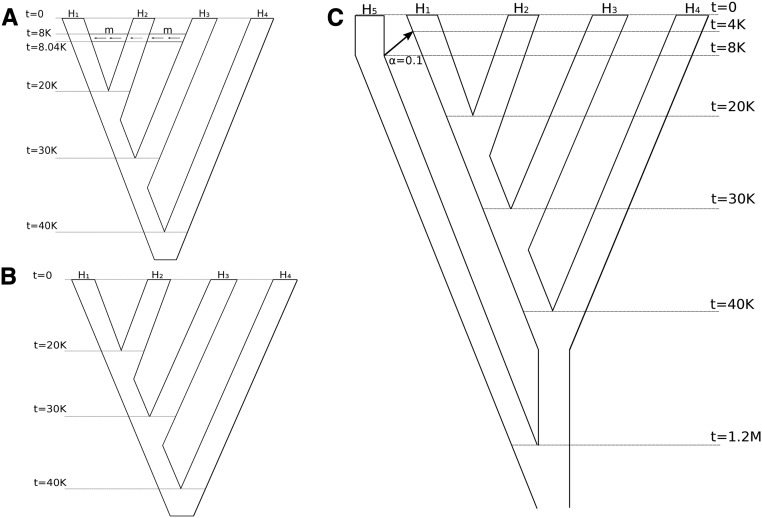
Simulated scenarios. (A) Simulation of a tree in which migration occurs from population $H_{3}$ to $H_{1}.$ The variable *m* is the (rescaled) migration rate varying from 0, 8, 16, 24, 32, 40, up to 280 with steps of size 20. Expressed as a percentage, the migration rate varies from 0, 0.02, 0.04, 0.06, 0.08, 0.1% up to 0.7%. Command: msms -N 10000 -ms 40 200 -I 4 10 10 10 10 0 -t 100 -r 100 1000 -em 0.2 3 1 $m -em 0.201 3 1 0 -ej 0.5 1 2 -ej 0.75 2 3 -ej 1 3 4. The same command line has been applied with the option -I 4 40 40 40 40 0 to generate populations of 20 diploid individuals, used to study the power of the method using subsets of 1, 2, 5, 10, and 20 individuals of such populations. (B) Simulation of a tree in which no migration occurs, but type-specific errors on some individuals provide a rejection when testing for correctness of the null hypothesis. Command: msms -N 10000 -ms 8 200 -I 4 2 2 2 2 0 -t 100 -r 100 1000 -ej 0.5 1 2 -ej 0.75 2 3 -ej 1 3 4. (C) Simulation of a tree in which $H_{5}$ admixes with $H_{1}$ with an instantaneous unidirectional admixture of rate α = 0.1. In this case, we expect the null hypothesis to be rejected since $H_{5}$ will alter the counts of ABBA and BABA patterns, but the alternative hypothesis does not involve gene flow with $H_{3}.$ Command: msms -N 10000 -ms 50 200 -I 5 10 10 10 10 10 0 -t 100 -r 100 1000 -es 0.1 1 0.9 -ej 0.2 6 5 -ej 0.25 1 2 -ej 0.5 2 3 -ej 0.75 3 4 -ej 30 4 5.

Different scenarios have been generated using the coalescent simulator msms (Ewing and Hermisson 2010) to reproduce the trees of Figure 2, A–C, in which times are in units of generations. Each topology has been simulated 100 times for a constant population size of $N_{e}=10^{4}.$ Mutation and recombination of the simulations are consistent with human data (Ewing and Hermisson 2010). Migrations and admixtures, respectively, for the scenarios of Figure 2, A and C, were simulated with specific options of msms. For each simulation, we generated 200 regions of size 5 Mb for each individual and considered only variable sites, except for the case of Figure 2B, where the null hypothesis was affected by type-specific errors on some of the individuals. We used a type-specific error of *e_A→G_* = 0.005 in populations $H_{1},H_{3}.$ The choice of the region size is compatible with the one estimated for applications with human genomes in Rasmussen *et al.* (2010). The regions are used by the jackknife estimator (Busing *et al.* 1999) to estimate the SD of the D-statistic accommodating the nonindependence of loci.

As a second step, the simulated genotypes from msms were given as inputs to msToGlf, a tool that is provided with ANGSD. Using msToGlf, it is possible to simulate NGS data from msms output files by generating the pileup files, which are used as input for ANGSD. As parameters for msToGlf, we set up the depth as the mean of a Poisson distribution, and we hardcoded the error rates in the program when necessary for the scenario in Figure 2B.

### Sequenced human populations

**Figure 3:**
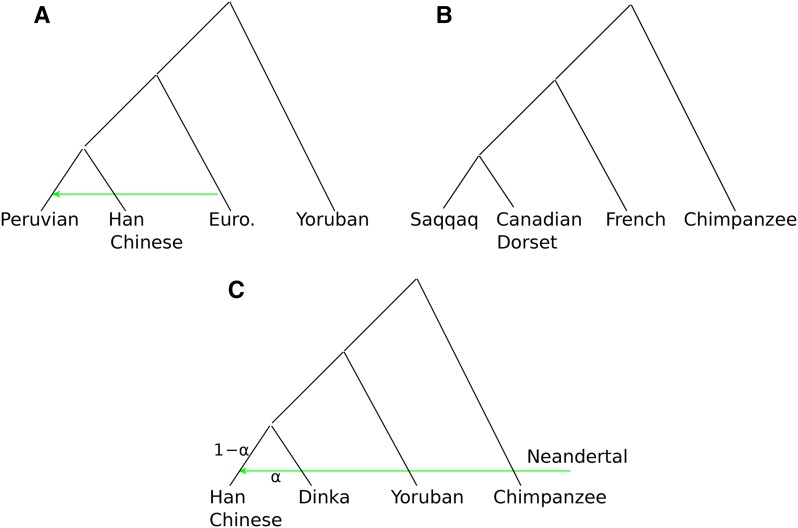
Real data scenarios. (A) Tree representing the southwestern European migration into the Americas during the European colonization. (B) Tree representing two independent migrations into northwestern Canada and Greenland. (C) Tree representing the presence of Neandertal genome in a modern non-African population, specifically the Han Chinese.

For the real data scenarios of Figure 3, A–C we used Illumina-sequenced individuals from several human populations. See Table 1 for an overview of the data. The depth of each individual has been calculated using the program doDepth of ANGSD. The Peruvian individuals used in our study were unadmixed with proportion ≥0.95. Estimation of the admixture proportions of these individuals was performed using ADMIXTURE (Alexander *et al.* 2009). In each individual, only the autosomal regions of all individuals were taken into consideration, and bases were filtered out according to a minimum base quality score of 20 and a mapping quality score of 30. Type-specific error estimates for the Saqqaq, Mi’kmaq, and French individuals were performed using the program doAncError of ANGSD, where the chimpanzee was used as the outgroup and the consensus sequence of human NA12778 as an error-free individual (see Figure S4 in File S1 for the bar plot of the estimates of the type-specific error).

**Table 1:** List of the genomes used in real data scenarios

Genome ID	Major Population Division	Depth	Reference
HG01923	Peruvian (PEL)	6.3×	Altshuler *et al.* (2010)
HG01974	Peruvian (PEL)	11.9×	Altshuler *et al.* (2010)
HG02150	Peruvian (PEL)	7.3×	Altshuler *et al.* (2010)
HG02259	Peruvian (PEL)	6.5×	Altshuler *et al.* (2010)
HG02266	Peruvian (PEL)	3.8×	Altshuler *et al.* (2010)
NA18526	Han Chinese (CHB)	6.6×	Altshuler *et al.* (2010)
NA18532	Han Chinese (CHB)	7.3×	Altshuler *et al.* (2010)
NA18537	Han Chinese (CHB)	2.9×	Altshuler *et al.* (2010)
NA18542	Han Chinese (CHB)	7.3×	Altshuler *et al.* (2010)
NA18545	Han Chinese (CHB)	6.2×	Altshuler *et al.* (2010)
NA06985	CEPH (CEU)	12.8×	Altshuler *et al.* (2010)
NA06994	CEPH (CEU)	5.5×	Altshuler *et al.* (2010)
NA07000	CEPH (CEU)	9.4×	Altshuler *et al.* (2010)
NA07056	CEPH (CEU)	4.9×	Altshuler *et al.* (2010)
NA07357	CEPH (CEU)	5.7×	Altshuler *et al.* (2010)
NA12778	CEPH (CEU)	6.9×	Altshuler *et al.* (2010)
NA18501	Yoruba (YRI)	6.4×	Altshuler *et al.* (2010)
NA18502	Yoruba (YRI)	4.9×	Altshuler *et al.* (2010)
NA18504	Yoruba (YRI)	10.1×	Altshuler *et al.* (2010)
NA18505	Yoruba (YRI)	6.1×	Altshuler *et al.* (2010)
NA18507	Yoruba (YRI)	3×	Altshuler *et al.* (2010)
HGDP00778	Han Chinese (CHB)	23.4×	International HapMap Consortium (2003)
DNK02	Dinka	25.8×	Meyer *et al.* (2012)
HGDP00927	Yoruban (YRI)	28×	International HapMap Consortium (2003)
AltaiNea	Neandertal	44.9×	Green *et al.* (2010)
pantro2	Chimpanzee	—	Kent *et al.* (2002)
saqqaq	Saqqaq	15.7×	Rasmussen *et al.* (2010)
MARC1492	Ancient Canadian Dorset	1.1×	Raghavan *et al.* (2014)
	(Mi’kmaq—New England)		
HGDP00521	French	23.8×	International HapMap Consortium (2003)

CEPH

Utah Resident with Northern and Western European Ancestry

### Data availability

The real data used is specified in Table 1. The simulated data has been produced using msms (Ewing and Hermisson 2010). The msms code for simulations is in the caption of Figure 2. From the output of msms, NGS pileup files were simulated with the tool msToGlf integrated in ANGSD (Nielsen *et al.* 2011). The one-sample D-statistic and the extended D-statistic implemented in this paper were performed on both real and simulated data with the program doAbbababa2 of ANGSD. ANGSD can be downloaded from https://github.com/ANGSD/angsd. A detailed guide including a tutorial for the program doAbbababa2 can be found at http://www.popgen.dk/angsd/index.php/Abbababa2.

## Results and Discussion

In the study of our results, we compare different implementations of the D-statistic on simulated and real scenarios. We briefly define as $D_{ext}$ the extended D-statistic that we implemented, $D_{1base}$ as the D-statistic calculated by sampling one sequenced base per locus (Green *et al.* 2010), and $D_{geno}$ the D-statistic calculated with equation (3) using the allele frequencies estimated from the true genotype (the true genotype is only available in the case of simulated data).

The D-statistic is computed on blocks of 5 Mb, to ensure that no block is subject to linkage disequilibrium from the other blocks, and that the number of loci in each block is large enough to make the D-statistic approach the approximation by a standard normal distribution (see Appendix 1). The use of blocks allows for estimation of a proper normalization constant for the D-statistic using the *m*-block jackknife method (Busing *et al.* 1999). The threshold for rejection of the null hypothesis is set to a p-value of 0.001, corresponding approximately to the two-tailed acceptance region [−3, 3].

The formula for calculating the D-statistic is given in equation (3). Its current implementations include those in Patterson *et al.* (2012) and Nielsen *et al.* (2011), with sampling of one base per locus from only one individual in each population. Such an implementation is computationally fast but has many drawbacks:

- when genomes are sequenced at low or medium depth (1–10×), sampling one base might lead to a process with high uncertainty;
- base transition errors might affect the sampling of the base, adding more uncertainty;
- only one individual per population is used;
- for a chosen individual from a population, the reads are not used to evaluate the D-statistic, but only to sample one base.

We have proposed a solution to these problems with the extended version of the D-statistic $D_{ext}$ implemented in ANGSD, and we will show in the following results how all the problems mentioned above are addressed.

### Comparison of power among the different methods

Using simulated and real data, we compare the different types of D-statistics to study their sensitivity to gene flow. We illustrate how the improved D-statistic $D_{ext}$ is not affected by the issues faced by the current D-statistic $D_{1base},$ and how it even reaches the performances of the D-statistic based on the true genotype $D_{geno}$ at a rather low sequencing depth.

**Figure 4:**
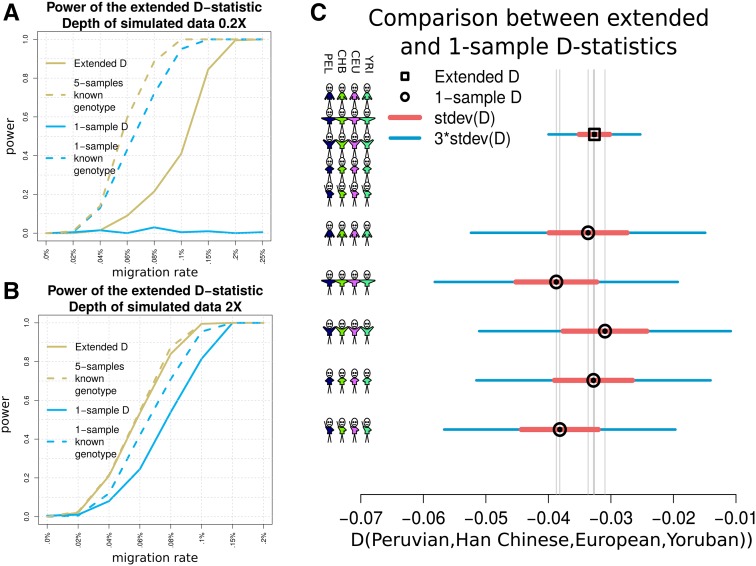
Detection of admixture and migration. (A and B) Rejection rate of the null hypothesis as a function of the migration rate in the tree $\left(\left(\left(H_{1},H_{2}\right)H_{3}\right)H_{4}\right),$ where a migration from $H_{3}$ to $H_{1}$ occurs. The yellow and blue solid lines represent, respectively, the power of the method related to $D_{ext}$ and $D_{1base}.$ The yellow dashed line represents the rejection rate when the genotypes of the five individuals in each population are known and thus equation (3) can be applied. The blue dashed line illustrates the power of the method when only one genome per population has known genotypes. $D_{ext}$ performs almost as well as knowing the true genotypes already with depth 2×. (C) Value of lack square) and values of $D_{1base}$ (black circles) using, respectively, five genomes per population and one from each population. Each D-statistic shows its associated SD multiplied by 1 and 3. On the left side of the graph, the stick men represent for each column the composition of the group by number of individuals.

To evaluate the power of the different methods, we first simulated NGS data based on coalescent simulations with mutation and recombination rates consistent with human populations (Ewing and Hermisson 2010). We simulated without sequencing error four populations with a varying amount of migration from $H_{3}$ to $H_{1}$ (see Figure 2A) and applied the D-statistic based on five individuals from each population for two different sequencing depths. Figure 4, A and B show the power of the methods for depths 0.2× and 2×. Here, the power is the rejection rate of the null hypothesis when there is a migration from $H_{3}$ to $H_{1}$ in the tree $\left(\left(\left(H_{1},H_{2}\right)H_{3}\right)H_{4}\right).$

The extended D-statistic proves to be effective in detecting gene flow even when the simulated depth is very low. For the scenario with sequencing depth 0.2×, $D_{1base}$ detects hardly any cases of migration from $H_{3},$ whereas $D_{ext}$ reacts with an acceptable rejection rate, even for a migration rate as low as m=0.15%. Of course, such a very low depth does not allow the D-statistic to perform as well as $D_{geno}.$ In the case of sequencing depth 2×, $D_{1base}$ does not always detect the alternative hypothesis and has also a considerable delay in terms of the migration rate necessary to do that, when compared with $D_{ext}.$ Furthermore, $D_{ext}$ follows almost exactly the behavior of the power related to $D_{geno}.$ This means that with a depth above 2× we can expect the D-statistic $D_{ext}$ to perform as well as knowing the exact genotypes of the data.

A deeper analysis to study the effects of using multiple individuals per group is illustrated in Figure S1 in File S1. Here, we simulated again the scenario with depth 0.2×, and compared the use of 1, 2, 5, 10, and 20 individuals per population. The graph shows that using multiple individuals increases the power of the method and at the same time decreases the SD of $D_{ext}.$

From 5000 simulations of the null hypothesis at depth 0.2×, we produced the quantile–quantile plot shown in Figure S2 in File S1. Here we can see that, despite us having simulated only 200 blocks of 5 Mb in length for each individual, the D-statistic already shows its asymptotic property of convergence to a standard normal distribution.

The powers of $D_{ext}$ and $D_{1base}$ are compared in a real data scenario using Illumina-sequenced modern human populations from the 1000 Genomes Project, with a varying sequencing depth in the range 3 to 13×. We specifically used Peruvian, European, Han Chinese, and African Yoruban individuals to form the tree (((Peruvian,Han Chinese)European)Yoruban) shown in Figure 3A. This scenario represents the southwestern European gene flow into the ancestors of the Native Americans (Raghavan *et al.* 2013). Each of the four populations consists of five sequenced individuals when evaluating $D_{ext},$ and a distinct one of those individuals when evaluating $D_{1base}$ five times (see Figure 4C). The extended D-statistic $D_{ext}$ has much lower SE, which corresponds to a smaller p-value than in the case of $D_{1base},$ and therefore a more significant rejection. See Table S1 in File S1 for a better comparison of the values of the different D-statistics.

It is worth underlining that the presence of structured populations might lead to false positives, because the structure is not considered in the model. If there is structure within $H_{1},H_{2},$ the properties of the D-statistic are preserved. However, if the population was structured prior to the split of *H_1_* and *H_2_*, then it will affect the D-statistic.

### Error impact and correction

Sequencing or genotyping errors are known to have a large impact on the D-statistic (Orlando *et al.* 2013). Using simulation, we show that if the type-specific error rates are known then we can correct the D-statistic accordingly. We simulate the tree under the null hypothesis. However, we add a base *A → G* error rate of 0.005 in populations $H_{1}$ and $H_{3}$ in order to alter the observed number of ABBA and BABA combination of alleles, leading to a possible rejection of the null hypothesis.

**Figure 5:**
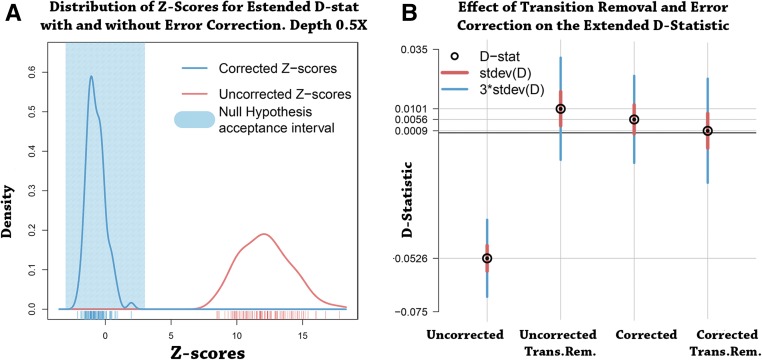
Effect of error estimation and correction. (A) Estimated distributions of the Z-scores related to $D_{ext}$ for the null hypothesis $\left(\left(\left(H_{1},H_{2}\right)H_{3}\right)H_{4}\right)$ in which $H_{1},H_{3}$ and $H_{2}$ have probabilities 0.005 and 0.01 of transition from base A, respectively. The blue polygon represents the interval where a Z-score would accept the null hypothesis. The red line represents the distribution of Z-scores before type-specific errors are corrected. In blue, we have the Z-scores after correction. (B) Values of $D_{ext}$ in four different cases for the tree (((Saqqaq,Dorset)French)chimpanzee). The black circles are the values of the uncorrected D-statistic, removal of ancient transitions, error correction, error correction and ancient transition removal. The red and blue lines represent the SD and the value they need to reach the threshold of |Z|=3, respectively.

The plot in Figure 5A represents the estimated distributions of the Z-scores related to $D_{ext}$ before and after error estimation and error correction, for 100 simulations of a tree $\left(\left(\left(H_{1},H_{2}\right)H_{3}\right)H_{4}\right)$ without any gene flow, where we have also introduced type-specific errors for transitions from allele A to another allele for the individuals in $H_{1},H_{2},H_{3}$ at different rates. The test statistic has high values owing to the error, whereas all simulations fall in the acceptance interval if we perform error correction.

The uncorrected D-statistic performs poorly because of the errors in the data that cause rejection of the null hypothesis in all simulations. It is remarkable to observe that $D_{ext}$ has good performance even at depth 0.5×. This means that even small error rates in the data make the D-statistic very susceptible to the rejection of *H_0_*. Therefore, we need to apply error correction to our data. The result is that the Z-scores fall into the acceptance threshold and the null hypothesis is fulfilled. The distribution of corrected Z-scores is not perfectly centered on 0 because of imperfect error correction.

The most obvious need for error correction in real applications is in the use of ancient genomes, which have large numbers of errors, especially transitions. To illustrate the effects of errors in real data and our ability to correct for them, we use two ancient genomes that contain high sequencing error rates owing to *post mortem* deamination. The tree (((Saqqaq,Dorset)French)chimpanzee) of Figure 3B illustrates the migrations to western Canada (Canadian Dorset Mi’kmaq genome) and southwestern Greenland (Saqqaq genome). Owing to the effects of deamination prior to sequencing (Rasmussen *et al.* 2010; Raghavan *et al.* 2014), the two ancient genomes have high type-specific error rates, as shown in Figure S4 and Table S2 in File S1. The error rates alter the counts of ABBA and BABA patterns, which bias the uncorrected D-statistic.

We expect the tree to be true under the null hypothesis, since Saqqaq and Dorset have a recent common ancestor (Raghavan *et al.* 2015). In Figure 5B, we compare the extended D-statistic $D_{ext}$ in four cases: first, using observed data; second, removing all transitions, which are related to most of the errors; third, applying error correction; and, last, combining error correction and transition removal. Note that the removal of transitions related to the pairs of alleles A,C and G,T is the current standard technique to avoid high error rates when calculating the D-statistic from damaged low-coverage data. The uncorrected D-statistic rejects the null hypothesis, whereas correction or transition removal gives a nonsignificant test. Error correction performs better than transition removal, providing a value of the D-statistic that is closer to 0 and has smaller SD. Table S3 in File S1 shows the values related to the four D-statistics in this scenario. Figure S5 in File S1 illustrates the effects of increasing and decreasing the removal of error for the base transitions *C* → *G* and *C* → *T* for one of the Saqqaq, Dorset, and French genomes. This corresponds to adding a value to the estimated error rate matrix of one of the individuals. Observe that the French individual is less affected by the addition or removal of error than the other two individuals. Moreover, all three individuals are more sensitive to the error rate in the case of transversion *C* → *T*.

### Correction for external introgression

We use simulations of a scenario with external introgression to verify the performance of correction for gene flow in restoring a four-population tree configuration that leads to the acceptance of the null hypothesis *H*_0_. In the simulation case, we know the value of α, that is, the amount of introgression; therefore, correction is possible. Thereafter, we use a known genetic relationship involving the Neandertal introgression into out-of-Africa modern individuals in Europe and Asia (Green *et al.* 2010; Wall *et al.* 2013) to correct for the effect of admixture. In addition, we show that, if we assume the absence of gene flow in the tree topology, we can estimate the amount of introgression and compare it with the estimation involving the original D-statistic tools.

**Figure 6:**
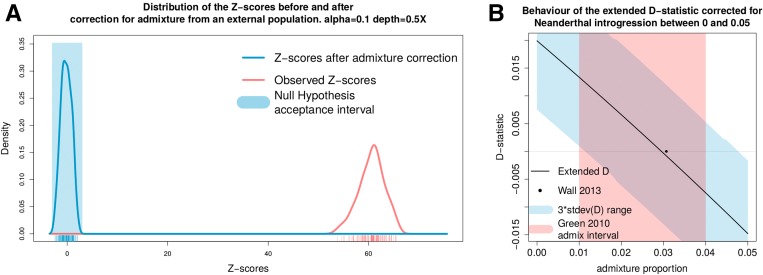
Effect of correction from external introgression. (A) Estimated distribution of the Z-scores related to $D_{ext}$ from the 100 simulations of the null hypothesis $\left(\left(\left(H_{1},H_{2}\right)H_{3}\right)H_{4}\right)$ with introgression of rate α = 0.1 from an external population $H_{5}$ into $H_{2}.$ The Z-scores of the observed tree are far off the acceptance interval because of the admixture from $H_{5}.$ Once the portion of genome from the external population is removed from $H_{2},$ the tree fulfills the null hypothesis and the Z-scores all fall in the acceptance interval defined by |Z|≤3. . (B) Behavior of the $D_{ext}$ of the tree (((Han Chinese,Dinka)Yoruban)chimpanzee) as a function of the admixture rate α used to correct for the introgression of the Neandertal population into the Han Chinese population. The red polygon is the interval in which (Green *et al.* 2010) estimates α to fall. The black dot coincides with the value of α = 0.0307 calculated by Wall *et al.* (2013) using the tree (((Han Chinese,Yoruban)Neandertal)chimpanzee), with SD 0.0049. The blue polygon is three times the SD of $D_{ext}.$ When $D_{ext}$ is 0, we estimate α = 0.03 with SD 0.0042.

For some species, there are introgression events from an external source, which can affect the D-statistic when performing tests for admixture among the species. We performed 100 simulations of the null hypothesis $\left(\left(\left(H_{1},H_{2}\right)H_{3}\right)H_{4}\right)$ of Figure 2C, for which an external population $H_{5}$ is admixed with $H_{2}$ with rate α = 0.1. The plot in Figure 6A shows the estimated distribution of the Z-scores related to the observed and admixture-corrected $D_{ext}.$ The observed D-statistic is positive and has Z-scores that reject the null hypothesis. Applying equation (7), we are able to remove the effect of gene flow from $H_{2}.$ The result of removal of the gene flow effect is that the estimated probabilities of ABBA and BABA combinations of alleles are altered, and the resulting calculated values of the D-statistic lead to acceptance of the null hypothesis *H*_0_.

For human populations, it is problematic to apply the D-statistics to both African and non-African populations because of ancient gene-flow from other hominids into non-Africans. Therefore, *H*_0_ might not be fulfilled for any tree $\left(\left(\left(H_{1},H_{2}\right)H_{3}\right)H_{4}\right)$, where an ingroup consists of both an African and a non-African population. This leads to rejection of the tree and to the natural conclusion that there is gene flow between $H_{3},H_{2}$ (respectively, $H_{3},H_{1}$). However, if there is known external admixture from a population $H_{5},$ it is possible to correct for admixture from this external contribution.

We illustrate the problem and our ability to correct for it using the tree shown in Figure 3C, which shows introgression of the Neandertal genome into the ancestors of the Han Chinese population. The correction is performed for the admixture proportion α in the range [0, 0.05] in steps of 0.01. The value of α for which $D_{ext}$ is closest to 0 might be considered as an estimate of the admixture rate. We chose these populations because we could compare our result with the estimate from previous studies of the same populations (Green *et al.* 2010; Wall *et al.* 2013). The study of Green *et al.* (2010) estimated α to be in the range [0.01, 0.04], whereas (Wall *et al.* 2013) estimated it as being α = 0.0307 with SD 0.0049. The result is shown in Figure 6B for the tree (((Han Chinese,Dinka)Yoruban)chimpanzee) for different admixture rates α used to correct for the introgression of the Neandertal population into the Han Chinese population. The red polygon is the interval in which α is estimated to be (Green *et al.* 2010). The black dot coincides with the value of α = 0.0307 calculated in Wall *et al.* (2013). The blue polygon is three times the SD of $D_{ext}.$ For almost the whole range of reported admixture proportions, the tree is not rejected after adjustment for admixture, indicating that the uncorrected D-statistic concluded the presence of gene flow. When $D_{ext}$ is 0, we estimate α = 0.03 with SD 0.0042, which is similar to previous estimates.

In the cases of both simulated and real data, we have thus been able to distinguish the case in which the alternative hypothesis is due to an external introgression and not to admixture from $H_{3}.$ In our simulations, the admixture correction seems not to suffer from the effect of drift, which is not modeled in the correction. In fact, the branch leading to $H_{5}$ splits 8000 generations in the past and admixes 4000 generations in the past on the branch leading to $H_{1}.$ Thus, there is a drift affecting gene frequencies of both the admixing and admixed populations.

In the case of real data, the exact amount of admixture α was not previously known. Therefore, we calculated the D-statistic for the tree (((Han Chinese,Dinka)Yoruban)chimpanzee) using admixture-corrected values of the probabilities of allele patterns, considering values of the admixture rate falling in the interval estimated in Green *et al.* (2010). Without admixture correction, the obvious conclusion would have been that for the tree (((Han Chinese,Dinka)Yoruban)chimpanzee) there is gene flow between the Yoruban and Dinka populations.

### Conclusions

In summary, we have implemented a different D-statistic that addresses the drawbacks of the current implementations of the D-statistic, but still preserves the approximation as a standard normal distribution (see Appendix 1) that allows for a statistical test. The extended D-statistic $D_{ext}$ allows for multiple individuals per population and, instead of sampling one base according to the estimated allele frequencies, uses all the available sequenced bases.

Using both simulations and real data we have shown that:

1. the extended D-statistic $D_{ext}$ has more power than the alternative methods, with an increased sensitivity to admixture events. Moreover, even without a large amount of data, the extended D-statistic shows a good asymptotic convergence and, therefore, a low false positive rate;
2. the performance of the extended D-statistic is the same as when the true genotype is known, for a depth of at least 2×;
3. we can accommodate type-specific errors to prevent an eventual wrong acceptance or rejection of the null hypothesis caused by error-affected allele frequencies. The error estimation and correction appear to be especially suited to the case of ancient genomes, where error rates might be high owing to chemical treatments prior to sequencing and degradation over time;
4. we can calculate the D-statistic after correcting for admixture from an external known population, such as in the case of Neandertal gene flow into the Han Chinese population.

The extended D-statistic $D_{ext}$ is especially effective compared with the standard D-statistic $D_{1base}$ when applied to data with low or variable depth, multiple individuals, and ancient DNA.

## Supplementary Material

Supplemental material is available online at www.g3journal.org/lookup/suppl/doi:10.1534/g3.117.300192/-/DC1.

## APPENDICES

The setup of the theoretical treatment consists of four sampled genomes representing four populations $H_{1},H_{2},H_{3},H_{4},$ for which we assume the relationship illustrated in Figure 1. Each genome is considered to have *M* di-allelic loci. We will consider the situation in which *M* grows to infinity. Each locus *i* consists of a certain number $n_{j}^{i}$ of alleles A and B, where j=1,2,3,4, is the index of the *j*th genome. Moreover, we assume independence between the loci.

Assume that at a locus *i* the allele frequencies in the four groups of individuals $x_{i}:=(x_{1}^{i},x_{2}^{i},x_{3}^{i},x_{4}^{i})$ follow a locus-dependent distribution $F_{i}\left(x\right),\;i=1,\ldots ,M$, and let ${\hat{x}}_{i}:=({\hat{x}}_{1}^{i},{\hat{x}}_{2}^{i},{\hat{x}}_{3}^{i},{\hat{x}}_{4}^{i})$ be an unbiased estimator of $x_{i}$ at locus *i*, such as the relative frequencies of the allele A in each population. The populations’ frequencies are considered to be a martingale process.

The null hypothesis that the tree of Figure 1 is correct can be rewritten as follows:

$$H_{0}:E\left[\left(x_{1}^{i}-x_{2}^{i}\right)\left(x_{3}^{i}-x_{4}^{i}\right)\right]=0,\;\text{for}\;i=1,\ldots ,M,$$

, where the expectation is done on the difference between the probabilities of ABBA and BABA events deduced in equations (1) and 2. Using the empirical frequencies as proxies for the expected values, we build the following normalized test statistic, also known as D-statistic:

$$D_{M}\;:=\;\frac{X_{\left(M\right)}}{Y_{\left(M\right)}}=\frac{\sum_{i=1}^{M}({\hat{x}}_{1}^{i}-{\hat{x}}_{2}^{i})({\hat{x}}_{3}^{i}-{\hat{x}}_{4}^{i})}{\sum_{i=1}^{M}({\hat{x}}_{1}^{i}+{\hat{x}}_{2}^{i}-2{\hat{x}}_{1}^{i}{\hat{x}}_{2}^{i})({\hat{x}}_{3}^{i}+{\hat{x}}_{4}^{i}-2{\hat{x}}_{3}^{i}{\hat{x}}_{4}^{i})}$$

where the values

$$X_{\left(M\right)}=\;\overset{M}{\underset{i=1}{\sum }}({\hat{x}}_{1}^{i}-{\hat{x}}_{2}^{i})({\hat{x}}_{3}^{i}-{\hat{x}}_{4}^{i})$$,

$$Y_{\left(M\right)}=\;\overset{M}{\underset{i=1}{\sum }}({\hat{x}}_{1}^{i}+{\hat{x}}_{2}^{i}-2{\hat{x}}_{1}^{i}{\hat{x}}_{2}^{i})({\hat{x}}_{3}^{i}+{\hat{x}}_{4}^{i}-2{\hat{x}}_{3}^{i}{\hat{x}}_{4}^{i})$$

are the numerator and denominator of the D-statistic, respectively.

### Appendix 1

#### Convergence of the D-statistic

In this paragraph we prove that the D-statistic defined as

$$D_{M}=\frac{X_{\left(M\right)}}{Y_{\left(M\right)}}$$

converges in distribution to a standard normal variable up to a constant.

Rewrite the numerator and denominator as

$$X_{\left(M\right)}=\;\overset{M}{\underset{i=1}{\sum }}X_{i}$$

$$Y_{\left(M\right)}=\;\overset{M}{\underset{i=1}{\sum }}Y_{i}$$

where the values $X_{i}$ and $Y_{i}$ are defined for each i=1,…,M by

$$X_{i}=({\hat{x}}_{1}^{i}-{\hat{x}}_{2}^{i})({\hat{x}}_{3}^{i}-{\hat{x}}_{4}^{i})$$

$$Y_{i}=({\hat{x}}_{1}^{i}+{\hat{x}}_{2}^{i}-2{\hat{x}}_{1}^{i}{\hat{x}}_{2}^{i})({\hat{x}}_{3}^{i}+{\hat{x}}_{4}^{i}-2{\hat{x}}_{3}^{i}{\hat{x}}_{4}^{i}))$$

Consider the series of independent variables $X_{i}$ in the numerator of $D_{M},$ having means $\mu_{i}.$ Every term $X_{i}$ of the numerator is an unbiased estimate of $\left(x_{1}^{i}-x_{2}^{i})(x_{3}^{i}-x_{4}^{i}\right)$, assuming the observed allele counts are binomially distributed (Reich *et al.* 2009). We show in the following proposition that every term of the numerator of the D-statistic has expectation $\mu_{i}=0$ for i=1, …, M by calculating the expectation of $\left(x_{1}^{i}-x_{2}^{i})(x_{3}^{i}-x_{4}^{i}\right)$.

**Theorem 1.** *Given the tree topology of* *Figure 1*, *it holds that* $E\left[\left(x_{1}^{1}-x_{2}^{i})(x_{3}^{1}-x_{4}^{i}\right)\right]=0$ *for* i=1, …, M.

*Proof*. Let $x_{1:2}^{i},$ $x_{1:3}^{i}$, and $x_{1:4}^{i}$ be the frequencies of the ancestral populations of $\left(x_{1}^{i},x_{2}^{i}\right)$, $\left(x_{1}^{i},x_{2}^{i},x_{3}^{i}\right)$ and the root of the tree, respectively, as illustrated in Figure 1. Let χ be the set of those three frequencies. Using the martingale properties of the frequencies, it follows that

$$E\left[\left(x_{1}^{i}-x_{2}^{i}\right)\left(x_{3}^{i}-x_{4}^{i}\right)\right]=E\left[E\left[\left(x_{1}^{i}-x_{2}^{i}\right)\left(x_{3}^{i}-x_{4}^{i}\right)\text{ X}\right]\right]$$

$$=E\left[E\left[x_{1}^{i}-x_{2}^{i}\text{ Χ}\right]E\left[x_{3}^{i}-x_{4}^{i}\text{ X}\right]\right]$$

$$=E\left[E\left[x_{1}^{i}-x_{2}^{i}\chi_{1:2}\right]E\left[x_{3}^{i}-x_{4}^{i}\text{ X}\right]\right]$$

$$=E\left[0\cdot E\left[x_{3}^{i}-x_{4}^{i}\text{ X}\right]\right]=0$$. (8)

Therefore $X_{i}$ has mean 0 for all i=1,…,M.

To prove convergence of the D-statistic for large *M* we assume the following:

1. Let ${\text{σ}}_{i}^{2}$ be the variance of every term $X_{i}.$ Denote with $v_{M}$ the sum $\overset{M}{\underset{i=1}{\sum }}\sigma_{i}^{2}$, then
  $$v_{M}\to \infty \text{ for }M\to \infty$$. (9)
2. Let $Y_{i},\;i=1,\ldots ,M$ be the series of independent variables in the denominator of $D_{M},$ having means $\gamma_{i}.$ Then
  1M∑i=1Mγi→γ for M→∞. (10)
3. Denote with ${\text{τ}}_{i}^{2}$ the variance of $Y_{i}.$ Then
  $$\frac{1}{M^{2}}\overset{M}{\underset{i=1}{\sum }}\tau_{i}^{2}\to \tau \text{ for }M\to \infty$$. (11)

If the numerator and denominator are sums of independent and identically distributed (IID) variables, conditions (9), (10), and (11) are fulfilled. In fact, if every term $X_{i}$ has variance ${\text{σ}}^{2}$, the sum of variances is $v_{M}=M\sigma^{2}$ and (9) holds. If every term $Y_{i}$ has mean and variance γ and ${\text{τ}}^{2}$, respectively, equation (10) is still valid because the arithmetic mean is done on identical values. Moreover, equation (11) holds because

$$\frac{1}{M^{2}}\overset{M}{\underset{i=1}{\sum }}\tau^{2}=\frac{1}{M}\tau^{2}$$,

which converges to zero for M→∞.

The convergence of the D-statistic $D_{M}$ is proved in steps, analyzing separately the numerator and the denominator. We begin by stating all the necessary theorems. First, we consider an extension of the central limit theorem (CLT) (Johnson 2004), which will be applied to the numerator $X_{\left(M\right)}.$ Subsequently, we state the law of large numbers (LLN) (Lamperti 1996) for not-IID variables that is used for the denominator $Y_{\left(M\right)}$ of the D-statistic. Thereafter, we enunciate one of the consequences of Slutsky’s theorem (Slutsky 1925; Pesaran 2015). The last step is a theorem for the convergence of the D-statistic, proved by invoking all the previous statements and applied to the specific case of the D-statistic.

**Theorem 2** (*CLT for independent and not identically distributed variables*). Let *${\left\{X_{i}\right\}}_{i=1}^{M}$* be a sequence of independent (but not necessarily identically distributed) variables with zero mean and variances ${\text{σ}}_{i}^{2}$. Define $v_{M}$ as $\overset{M}{\underset{i=1}{\sum }}\sigma_{i}^{2}$ Consider the following quantity

$${\text{Λ}}_{\epsilon }\left(M\right):=\overset{M}{\underset{i=1}{\sum }}E\left[{\left(\frac{{\text{X}}_{i}}{\sqrt{v_{M}}}\right)}^{2}I\left(\left|\frac{{\text{X}}_{i}}{\sqrt{v_{M}}}\right|\right)\geq \;\epsilon \right]$$

where I(⋅) defines the indicator function. If for any ϵ>0 it holds that $\underset{M\to \infty }{\lim}{\text{Λ}}_{\epsilon }\left(M\right)=0$, then the normalized sum $U_{M}=\overset{M}{\underset{i=1}{\sum }}{\text{X}}_{i}/\sqrt{v_{M}}$ converges in distribution to a standard normal N(0,1).

**Theorem 3** (*LLN for independent and not identically distributed variables*). Let ${\left\{Y_{i}\right\}}_{i=1}^{M}$ be a sequence of uncorrelated random variables. Define *${\bar{Y}}_{M}$* as the empirical average $\frac{1}{M}\overset{M}{\underset{i=1}{\sum }}Y_{i}$. Denote with ${\text{γ}}_{i}$ and ${\text{τ}}_{i}^{2}$ the expectation and variance of each variable. If conditions (10) and (11) are fulfilled, then for each ϵ<0

$$\underset{M\to \infty }{\lim}\mathbb{P}\left(\left|{\hat{Y}}_{M}-\frac{1}{M}\overset{M}{\underset{i=1}{\sum }}\gamma_{i}\right|\;\geq \;\epsilon \right)=0$$.

Equivalently, the empirical average *${\bar{Y}}_{M}$* converges in probability to $\underset{M\to \infty }{\text{lim}}\frac{1}{M}\gamma_{i}=\gamma .$

**Theorem 4** (*Slutsky’s theorem*). Let *$X_{\left(M\right)}$* and *$Y_{\left(M\right)}$* be two sums of not-IID random variables. If the former converges in distribution to *X* and the latter converges in probability to a constant γ for M→∞, then the ratio *$X_{\left(M\right)}/Y_{\left(M\right)}$* converges in distribution to *X*/γ.

The last step is a theorem for the convergence of the D-statistic, proved by invoking all the previous statements, applied to the specific case of the D-statistic.

**Theorem 5** (*Convergence in distribution of the D-statistic*). Consider the D-statistic defined by

$$D_{n}=\frac{X_{\left(M\right)}}{Y_{\left(M\right)}}=\frac{\sum_{i=1}^{M}X_{i}}{\sum_{i=1}^{M}Y_{i}}\in [-1,+1]$$,

where numerator and denominator are sum of independent (but not necessarily identically distributed) variables. Under the assumptions of (9), (10), and (11), the D-statistic converges in distribution to a standard normal if rescaled by the constant:

$$c_{M}D_{M}\overset{d}{\to }N(0,1)\text{ for }M\to \infty$$.

The arrow denotes the convergence in distribution, and *$c_{M}$* is defined as

$$c_{M}:=\gamma \frac{M}{\sqrt{v_{M}}}$$.

Here *$v_{M}$* is the sum of the variances of the first *M* terms of the numerator, and γ is the convergence value of the arithmetic mean of the denominator’s expectations for M→∞.

*Proof*. First consider Theorem 2 applied to the rescaled numerator $U_{M}=X_{\left(M\right)}/\sqrt{v_{M}}.$ It is necessary to prove that for any ϵ>0 it holds that $\underset{M\to \infty }{\lim}{\text{Λ}}_{\epsilon }\left(M\right)=0$ to ensure the convergence in distribution. First observe that $|X_{i}|\;\leq 1$ for any index *i*. Consequently we have the inequality

$${\text{Λ}}_{\epsilon }\left(M\right)\leq {\left(\frac{1}{\sqrt{v_{M}}}\right)}^{2}\overset{M}{\underset{i=1}{\sum }}E\left[I\left(\left|\frac{1}{\sqrt{v_{M}}}\right|\geq \epsilon \right)\right]=\;\frac{1}{v_{M}}\mathbb{P}\left(\left|X_{i}\right|\geq \epsilon \sqrt{v_{M}}\right)\leq \frac{1}{v_{M}}\frac{E\left[X_{i}\right]}{\epsilon \sqrt{v_{M}}}$$,

where Markov’s inequality is applied to the last line of the equation. Thus $U_{M}$ converges in distribution to a standard normal *N*(0,1).

Since conditions (10) and (11) are fulfilled by assumption, it is possible to invoke Theorem 3 to state that the empirical average of the denominator $Y_{\left(M\right)}/M$ converges in probability to a constant γ, which is positive since every term of the denominator is positive.

Finally, we apply Theorem 4 using the proper constants that follow from Theorems 2 and 3 applied to the numerator and denominator, respectively. We proved that the sum $X_{\left(M\right)}/\sqrt{v_{M}}$ converges in distribution to a standard normal N(0,1) and $Y_{\left(M\right)}/M$ converges in probability to the constant γ, which is the limit of the arithmetic mean of equation 10. Thus the ratio

$$\frac{M}{\sqrt{v_{M}}}\frac{X_{\left(M\right)}}{Y_{\left(M\right)}}$$

converges in distribution to a Gaussian $N\left(0,{\sqrt{\gamma }}^{-1}\right).$ The convergence in distribution of $D_{M}$ to a standard normal variable is accomplished by rescaling by the following multiplicative constant

$$c_{M}=\gamma \frac{\sqrt{v_{M}}}{M}.$$

The results of this proof apply also in the following cases of the D-statistic.

1. The original D-statistic $D_{M}$ calculated by sampling a single base at each site from the available reads (Green *et al.* 2010) to estimate the sampling probabilities. In this case every term on the numerator has possible values −1, 0, +1. Each population frequency $x_{j}^{i}$ is parameter of a binomial distribution $Bin\left(1,x_{j}^{i}\right),$ and is estimated by the frequency of the observed base A at locus *i* in population *j*,
2. The D-statistic is evaluated using the estimated population frequencies $q_{j}^{i}$ defined in equation 4 for multiple individuals in a population (see Appendix 2). In fact, the estimator for multiple individuals is still an unbiased estimate for the population frequency (Li *et al.* 2010), therefore every term of the numerator is still an unbiased estimate for the difference between the probabilities of ABBA and BABA events.
3. The D-statistic is evaluated only over loci with allele frequency $x_{4}=1$ for population $H_{4}.$ This special case of D-statistic has been used, for example, to assess the presence of gene flow from the Neandertal population into modern out-of-Africa individuals, setting a chimpanzee as the outgroup, and considering only loci where the outgroup showed uniquely allele A (Green *et al.* 2010). In fact, Theorem 1 still holds because in equation (8) the term $E\left[x_{1}^{i}-x_{2}^{i}|x_{1:2}\right]$ is zero, independently of which values $x_{4}^{i}$ assumes.

### Appendix 2

#### Multiple genomes

We assume a di-allelic model with alleles A and B and the four populations $H_{1},H_{2},H_{3},H_{4}$ that each consist of a number of distinct individuals $N_{j},\;j=1,2,3,4,$ where *j* indexes the populations. Given the allele frequency $x_{j}^{i},\;j=1,2,3,4,$ at locus *i*, we model the observed data as independent binomial trials with parameters $n_{j}^{i}$ and $x_{j}^{i}$ for j=1,2,3,4, where $n_{j}^{i}$ is the number of trials. One possible unbiased estimator of the population frequency is

$${\hat{x}}_{j}^{i}:=\frac{n_{j}^{i,A}}{n_{j}^{i}},$$

where $n_{j}^{i,A}$ is the total number of As and $n_{j}^{i}$ the total number of bases observed for the selected population and locus.

For locus *i* denote the allele frequency of individual ℓ in population *j* as $x_{j,\ell }^{i}.$ We use as its unbiased estimator

$${\hat{x}}_{jl}^{i}:=\frac{n_{jl}^{i,A}}{n_{j}^{i}}$$

namely the ratio between the number of observed As and the total number of observed alleles at locus *i* in genome ℓ. The idea is to condense all the quantities ${\hat{x}}_{j}^{i}$ into a single value ${\hat{q}}_{j}^{i}\;$ that minimizes the variance of the sum of the estimated individuals’ frequencies with respect to a set of normalized weights

$${\left\{w_{j,l}^{i}\right\}}_{l=1}^{N_{h}},\overset{N_{h}}{\underset{l=1}{\sum }}w_{j,l}^{i}=1$$

such that

$${\hat{q}}_{j}^{i}:=\overset{N_{h}}{\underset{l=1}{\sum }}w_{j,l}^{i}\cdot {\hat{x}}_{jl}^{i}$$.

The estimated population frequency ${\hat{q}}_{j}^{i}\;$ is an unbiased estimator of the frequency of population *j* at the *i*th locus (Li *et al.* 2010). The aim of the weight estimate is to determine the set of weights that minimizes the variance of ${\hat{q}}_{j}^{i}$. To do this, we first determine the variance of each individual’s frequency.

Consider a genome *l* in population *j*. We approximate the frequency estimator of genome *l* in population *j*, namely ${\hat{x}}_{jl}^{i}$, defining

$$Y_{j,l}^{i}:=\frac{\sum_{m=1}^{n_{j,l}^{i}}I_{m}}{n_{j,l}^{i}},$$

where $n_{j,\ell }^{i}$ is the total number of reads for individual *l* and $I_{m}∼Bin(1,x_{j}^{i})$ for $m=1,...,\;n_{j,l}^{i}$. Note that the binomial variables are parametrized by $x_{j}^{i}$ and not by $x_{j,\ell }^{i}.$ The variance of $Y_{j,\ell }^{i}$ is

$$V\left[Y_{j,l}^{i}\right]=\frac{1}{{\left(n_{j,l}^{i}\right)}^{2}}\left(\overset{n_{j,l}^{i}}{\underset{m=1}{\sum }}V[I_{m}]+2\overset{n_{j,l}^{i}}{\underset{r<t}{\sum }}Cov[I_{r},I_{t}]\right).\;$$ (12)

The variance of the indicator function $I_{m}$is

$$V\left[I_{m}\right]=x_{j}^{i}\left(1-\;x_{j}^{i}\right).$$

It remains to find the covariance

$$Cov\left[I_{r},I_{t}\right]=E\left[I_{r}I_{t}\right]-E\left[I_{r}\right]E\left[I_{t}\right]=E\left[I_{r}I_{t}\right]-\;x_{j}^{i^{2}}$$,

where, marginalizing on the underlying genotype *G* and assuming Hardy–Weinberg equilibrium, it follows that

$$E\left[I_{r}I_{t}\right]=\;\underset{g\;\in \left\{AA,AB,BB\right\}}{\sum }\mathbb{P}\left(I_{r}I_{t}=1,\;G=g\right)=\mathbb{P}\left(I_{r}I_{t}=1\;|\;G=AA\right)\mathbb{P}\left(G=AA\right)+2\mathbb{P}\left(I_{r}I_{t}=1\;|\;G=\;AB\right)\left(G=AB\right)+\mathbb{P}\left(I_{r}I_{t}=1\;|\;G=\;BB\right)\mathbb{P}\left(G=BB\right)$$

$$=0+\;\frac{1}{2}\cdot \frac{1}{2}\cdot 2x_{j}^{i}\left(1-x_{j}^{i}\right)+1\cdot x_{j}^{i^{2}}=\;\frac{1}{2}x_{j}^{i}\left(1-\;x_{j}^{i}\right)+x_{j}^{i^{2}}.$$

Considering that the sum over r<t in equation (12) is made over $1/2n_{j,\ell }^{i}\left(n_{j,\ell }^{i}-1\right)$ equal expectations, we can write

$$V\left[Y_{j,l}^{i}\right]=\frac{1}{{\left(n_{j,l}^{i}\right)}^{2}}\left[n_{j,l}^{i}x\left(1-x\right)+2\frac{n_{j,l}^{i}\left(n_{j,l}^{i}-1\right)}{2}\frac{1}{2}x_{j}^{i}\left(1-x_{j}^{i}\right)\right]=\frac{1}{{\left(n_{j,l}^{i}\right)}^{2}}\left[n_{j,l}^{i}x_{j}^{i}\left(1-x_{j}^{i}\right)+2\frac{n_{j,l}^{i}\left(n_{j,l}^{i}-1\right)}{2}\frac{1}{2}x_{j}^{i}\left(1-x_{j}^{i}\right)\right]=\;\frac{n_{j,l}^{i}+1}{2n_{j,l}^{i}}x_{j}^{i}\left(1-x_{j}^{i}\right)=R_{j,l}^{i}x_{j}^{i}\left(1-x_{j}^{i}\right),$$

where for practical purposes we have defined, for each *l*th individual, $R_{j,\ell }^{i}$ as the ratio

$$\frac{n_{j,\ell }^{i}+1}{2n_{j,\ell }^{i}}.$$

Consider at this point the approximation of the variance of the weighted “pseudo-individual,” having estimated frequency ${\hat{q}}_{j}^{i}:=\;\overset{N_{j}}{\underset{l=1}{\sum }}w_{j,l}^{i}\cdot {\hat{x}}_{j,l}^{i}$.

$$V\left[{\hat{x}}_{j}^{i}\right]=\overset{N_{j}}{\underset{l=1}{\sum }}{\left(w_{j,l}^{i}\right)}^{2}V[{\hat{x}}_{j,l}^{i}]\approx \overset{N_{j}}{\underset{l=1}{\sum }}{\left(w_{j,l}^{i}\right)}^{2}V\left[Y_{j,l}^{i}\right].\;$$ (13)

Our objective is to perform a Lagrange-constrained optimization with respect to the weights, being sure to find a minimum since equation (13), as function of the weights, is convex. This is easily done as the Lagrange-parametrized function is

$$L\left(w_{j,1:N_{j}}^{i},\text{λ}\right)=\overset{N_{j}}{\underset{l=1}{\sum }}{\left(w_{j,l}^{i}\right)}^{2}x_{j}^{i}\left(1-x_{j}^{i}\right)R_{j,l}^{i}-\lambda \left(\overset{N_{j}}{\underset{l=1}{\sum }}w_{j,l}^{i}-1\right)$$

and it originates a linear system of equations of the form

$$2\cdot w_{j,1}^{i}\cdot x_{j}^{i}\left(1-x_{j}^{i}\right)R_{j,1}^{i}-\;\lambda =0$$

⋮ ⋮ = ⋮

$$2\cdot w_{j,N_{j}}^{i}\cdot x_{j}^{i}\left(1-x_{j}^{i}\right)R_{j,N_{j}}^{i}-\;\lambda =0$$

$$\overset{N_{j}}{\underset{l=1}{\sum }}w_{j,l}^{i}-1=0$$

whose solution provides us with the minimum values of the weights as follows $\forall l\in \{1,\;\ldots ,\;N_{j}\}$:

$$w_{j,l}^{i}=\;\frac{\prod_{m=1,\;m\neq l}^{N_{j}}R_{j,m}^{i}}{\sum_{k=1}^{N_{j}}\prod_{m=1,\;m\neq k}^{N_{j}}R_{j,m}^{i}}=\frac{{\left(R_{j,l}^{i}\right)}^{-1}}{\sum_{k=1}^{N_{j}}{\left(R_{j,k}^{i}\right)}^{-1}}$$

### Appendix 3

#### Error estimation and correction

Estimation of the type-specific errors follows the supplemental material of Orlando *et al.* (2013). Assume having one observed sequenced individual affected by base transition errors. This individual has an associated 4 × 4 error matrix e, such that the entry e(a,b) is the probability of observing a base of type *b* when the true base is of type *a*. Consider the tree ((T,R),O), in which the leaves are sequenced genomes affected by type-specific errors (T), an individual without errors, used as reference for the error correction (R), and an outgroup individual (O).

Assume that loci are independent and that the errors between pairs of alleles are independent given a base *o* in the outgroup and the error matrix e. Then the likelihood of the base *t* in the observed individual can be decomposed as a product through the loci:

$$\mathbb{P}\left(T=t\;|\;O=o,\;e\right)=\overset{M}{\underset{i=1}{\prod }}\mathbb{P}\left(T=t\;|\;O=o,\;e\right)$$

Marginalize any *i*th factor of the above equation over the true alleles before error $g_{i}\in \left\{A,C,G,T\right\}\;$ of the underlying true genotype:

$$\mathbb{P}\left(T=t\;|O=o,\;e\right)=\underset{g\in \{A,C,G,T\}}{\sum }\mathbb{P}\left(T_{i}=t_{i}\;|\;G_{i}=g_{i},\;O_{i}=o_{i},\;e\right)$$

$$=\underset{g\in \{A,C,G,T\}}{\sum }\mathbb{P}\left(T_{i}=t_{i}\;|\;G_{i}=g_{i},\;O_{i}=o_{i},\;e\right)\mathbb{P}\left(G_{i}=g_{i}|\;O_{i}=o_{i}\right)$$

$$=\underset{g\in \{A,C,G,T\}}{\sum }e(g_{i},t_{i})\mathbb{P}\left(G_{i}=g_{i}\;|\;O_{i}=o_{i}\right),\;$$

where the true genotype $g_{i}$ is independent of the error rates for each i=1,…,M. One can approximate the probability of observing $g_{i}$ conditionally to $o_{i}$ with the relative frequency of the base $g_{i}$ in the error-free individual *R*, for loci where the outgroup is $o_{i},$ that is:

$$\mathbb{P}\left(G_{i}=\;g_{i}|\;O_{i}=o_{i}\right)=\mathbb{P}\left(R_{i}=\;g_{i}\;|\;O_{i}=o_{i}\right).$$

It is possible to perform a maximum likelihood estimation by numerical optimization to obtain an estimate of the error matrix. Note that every entry $e\left(g_{i},t_{i}\right)$ is the same over all loci.

The rationale behind the error correction is that the count of each base in the genomes *T* and *R* should be the same, otherwise an excess of counts in *T* is due to error. This approach to error estimation has been applied in Orlando *et al.* (2013) to study type-specific errors in ancient horses’ genomes.

Assume that the error matrix $e_{l}$ has been estimated for every individual *l* in each *j*th group. For a specific genome *l* we have the following equation for each locus *i*

$$\mathbb{P}\left(T_{i}=t_{i}\;|\;e_{l}\right)=\mathbb{P}\left(T_{i}=t_{i}\;|\;e_{l},\;G\;\to \;t_{i}\right)e_{l}\left(t_{i},t_{i}\right)+\;\underset{{\hat{t}}_{i}\neq t_{i}}{\sum }\mathbb{P}\left(T_{i}=t_{i}\;|\;e_{l},\;G=\;{\hat{t}}_{i}\right)e_{l}\left(t_{i},t_{i}\right)$$.

The same equation can be expressed in matrix form as follows:

$$p_{T}^{i}=e_{\ell }p_{G}^{i},$$

where $p_{T}^{i}$ and $p_{G}^{i}$ are the vectors of probabilities of observing alleles at locus *i* in the T and R genomes, respectively. If the error matrix $e_{l}$ is invertible, we can find the error-corrected allele frequencies as

$$p_{G}^{i}=e_{\ell }^{-1}p_{T}^{i}.$$ (14)

The correction performed in equation (14) makes the estimated allele frequencies unbiased. The unbiasedness allows the numerator of the D-statistic to have mean zero, and makes the D-statistic calculated with error-corrected frequencies convergent to a standard normal distribution (see Appendix 1). In fact, consider for a certain locus the di-allelic scenario with alleles A and B. Let *n* be the number of observed bases. The number of alleles A in the absence of errors is

m∼Bin(n,x),

where *x* is the population frequency. Let $\epsilon_{A,B}$ and $\epsilon_{B,A}$ be the probabilities of having a transition from A to B and from B to A, respectively. Then the total number of observed A alleles is given by the sum of the two following variables:

$$m_{0}∼Bin(m,\;1-\;\epsilon_{A,B})$$,

$$m_{1}∼Bin(n-\;m,\epsilon_{A,B})$$.

The expected population frequency is given by

$$\frac{1}{n}E\left[m_{0}+m_{1}\right]=\frac{1}{n}E\left[E\left[m_{0}|m\right]\right]+\frac{1}{n}E\left[E\left[m_{1}|m\right]\right]$$

$$=x\left(1-\epsilon_{A,B}\right)+\left(1-x\right)\epsilon_{B,A}.$$

The error matrix and its inverse for the di-allelic case are expressed as follows:

$$e=\left[\begin{array}{cc} 1-\;\epsilon_{A,B} & \epsilon_{B,A}\\ \epsilon_{A,B} & 1-\;\epsilon_{B,A} \end{array}\right],e^{-1}=\frac{1}{C}\left[\begin{array}{cc} 1-\;\epsilon_{B,A} & -\epsilon_{B,A}\\ -\epsilon_{A,B} & 1-\;\epsilon_{A,B} \end{array}\right],\;$$

where $C=\left(1-\;\epsilon_{A,B}\right)\left(1-\;\epsilon_{B,A}\right)-\;\epsilon_{A,B}\epsilon_{B,A}$ is the constant arising from the inversion of a 2 × 2 matrix. The formula in equation (14) is rewritten as

$$\left[\begin{array}{c} \hat{x}\\ 1-\;\hat{x} \end{array}\right]=\frac{1}{C}\left[\begin{array}{cc} 1-\;\epsilon_{B,A} & -\epsilon_{B,A}\\ -\epsilon_{A,B} & 1-\;\epsilon_{A,B} \end{array}\right]\left[\begin{array}{c} \hat{z}\\ 1-\;\hat{z} \end{array}\right],\;$$ (15)

where $\hat{x}$ is the estimator of the error-corrected population frequency, and $\hat{z}\;$ is the estimated population frequency prior to error correction:

$$\hat{z}=\frac{m_{0}+m_{1}\;}{n}.$$

From equation (15) it is possible to deduce the following equality:

$$E\left[\hat{x}\right]=\frac{1}{C}\left(1-\;\epsilon_{B,A}\right)E\left[\hat{z}\right]-\frac{1}{C}\left(1-\;E\left[\hat{z}\right]\right)\epsilon_{B,A}=\frac{1}{C}x\left(1-\epsilon_{B,A}-\epsilon_{A,B}\right)=x.$$

This proves that the error-corrected estimators of the allele frequencies are again unbiased; therefore, calculating the D-statistic using error-corrected allele frequencies leaves the convergence results unchanged.
