# Supplementary material for: Powerful Inference with the D-Statistic on Low-Coverage Whole-Genome Data
Source: G3 (Bethesda). 2017 Dec 1;8(2):551–66. doi: 10.1534/g3.117.300192 (PMC5919751; doi:10.1534/g3.117.300192)

Supplemental Material.

The Supplemental Material contains two tables with numeric results related to a real data scenario, and five figures regarding the power of the method, the asymptotic behaviour of D_{ext}, the estimates of type-specific errors, the behaviour of the D-statistic and the correction for external introgression.

Table S1. European Introgression into Native American Individuals. The table contains the values of the different types of D-statistics used to create the plot of Figure 4C, reporting the D-statistic for the tree (((PEL,CHB)CEU)YRI). The first column denote if we are illustrating either the extended D-statistic, D_{ext}, or the D-statistic that uses a sampled base, D_{1base}. The column denoted by D is the D-statistic over all blocks of loci, used to estimate the standard deviation (third column) by bootstrapping. The Z-score represents the D-statistic normalized by its standard deviation. The last column represents the ratio between the estimated standard deviations of D_{1base} and D_{ext}.

D-statistic	D	stdev(D)	Z-score	$\frac{\sigma_{1base}}{\sigma_{ext}}$
D_{ext}	-0.032638	0.002449	-13.114101	-
D_{1base}	-0.038171	0.006164	-6.223641	2.51
D_{1base}	-0.032786	0.006244	-5.253267	2.54
D_{1base}	-0.030950	0.006708	-4.602315	2.74
D_{1base}	-0.038730	0.006480	-5.999972	2.64
D_{1base}	-0.033640	0.006244	-5.353646	2.55

Table S2. Estimated Error Rates. Estimated type-specific error rates for the ancient individuals Saqqaq and Canadian Dorset Mi'kmaq used in the tree of Figure 3B.

Individual	$A \to C$	$A \to G$	$A \to T$	$C \to A$	$C \to G$	$C \to T$
Saqqaq	1.90e-04	6.08e-04	3.27e-04	7.52e-04	1.22e-04	6.32e-04
Dorset	8.86e-05	1.15e-03	1.62e-04	2.04e-04	8.52e-05	5.22e-03
	$G \to A$	$G \to C$	$G \to T$	$T \to A$	$T \to C$	$T \to G$
Saqqaq	6.35e-04	1.26e-04	7.52e-04	3.28e-04	6.08e-04	1.91e-04
Dorset	5.21e-03	9.01e-05	2.06e-04	1.64e-04	1.15e-03	9.04e-05

Table S3. Extended D-Statistic in Real Data Scenario with Ancient Genomes. Table comparing the extended D-statistic with the application of error correction and/or transition removal for the tree of Figure 5B, where the ancient individuals Saqqaq and Canadian Dorset Mi'kmaq are affected by high type-specific error rates.

Correction	D_{ext}	$sd(D_{ext})$	$Z - score$	$p - value$
None	-5.26e-2	5.4e-3	-9.81	0
Trans.Rem.	1.01e-2	7.1e-3	1.41	1.57e-1
Error.Corr.	5.64e-3	6.1e-3	0.93	3.51e-1
Err.Corr & Tr.Rem	8.77e-4	7.3e-3	0.12	9.04e-1

Figure S1. Effect of the number of individuals per population in detecting admixture. Results from the simulation of the scenario of Figure 2A, subject to a migration from H_3 to H_1, using either 1, 2, 5, 10 or 20 individuals per population sequenced at depth 0.2X. (A) Power of the extended D-statistic for increasing values of the number of individuals per group. (B) The value of the standard deviation of D_{ext} for different number of individuals per population.

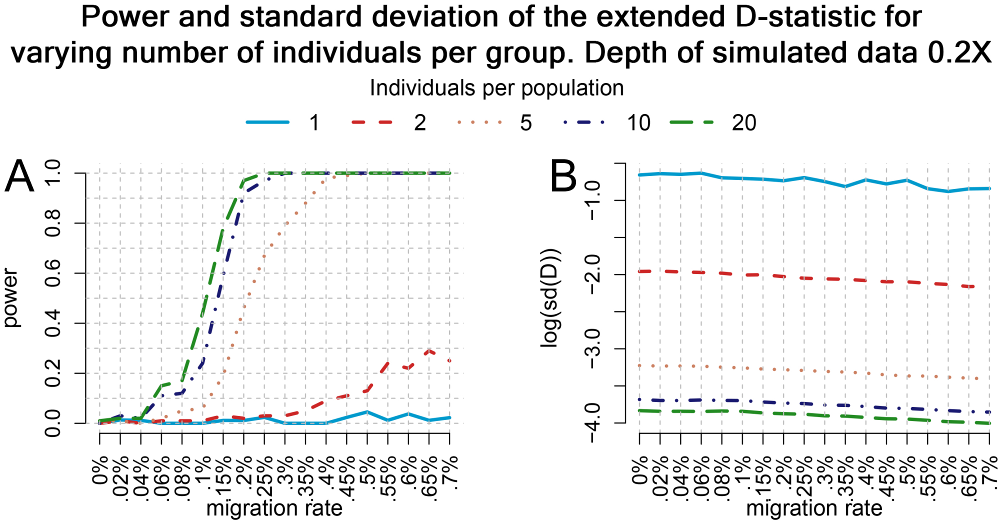

Figure S2. Asymptotic convergence of the extended D-statistic. QQ-plot of the observed log-pvalues from 5000 simulations of the null hypothesis of Figure 2B, where we have used 5 individuals per population and depth 2X. Each individual has 200 regions of length 5Mb. Despite that, the extended D-statistic D_{ext} shows already good properties of asymptotic convergence to the standard normal, with a slight problem due to few extreme pvalues.

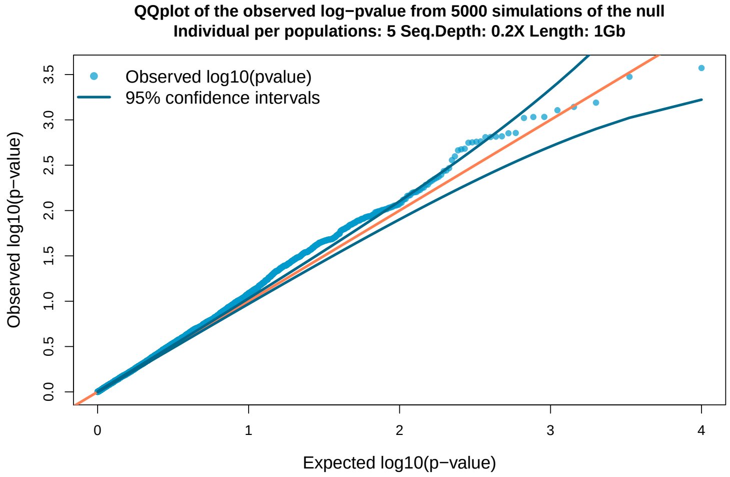

Figure S3. Subtrees of interest in a scenario subject to external introgression. (A) Case of a 4-population tree subject to introgression from an external population H_5. Consider H_2 being the population subject to introgression from H_5. (B) The subtree $T_{1:4}$ includes the 4-population tree excluding the admixing population. (C) The subtree T_{out} replaces the admixed population with the population source of introgression. (D) The subtree T_{un}, where H_2' represents H_2 when it has not yet undergone admixture, reflects the null hypothesis of correctness for the genetic relationship between four populations.

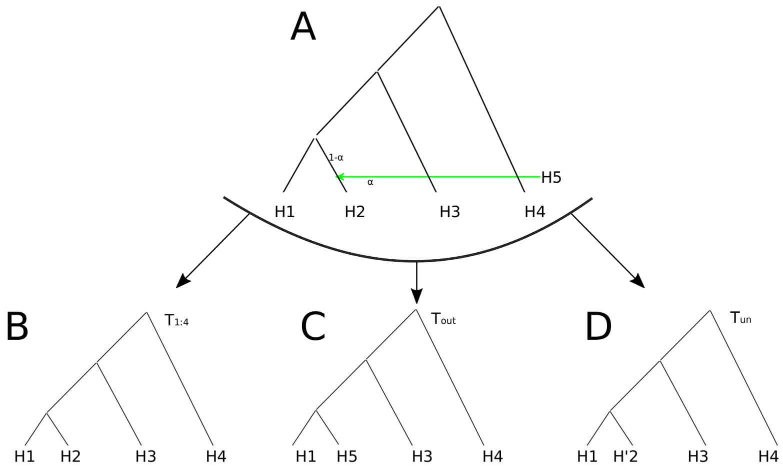

Figure S4. Estimates of Type-Specific Errors for Ancient Genomes. Estimated type-specific error rates for the Saqqaq, Mi'qmak and French genomes of the real data scenario illustrated in Fig 4B.

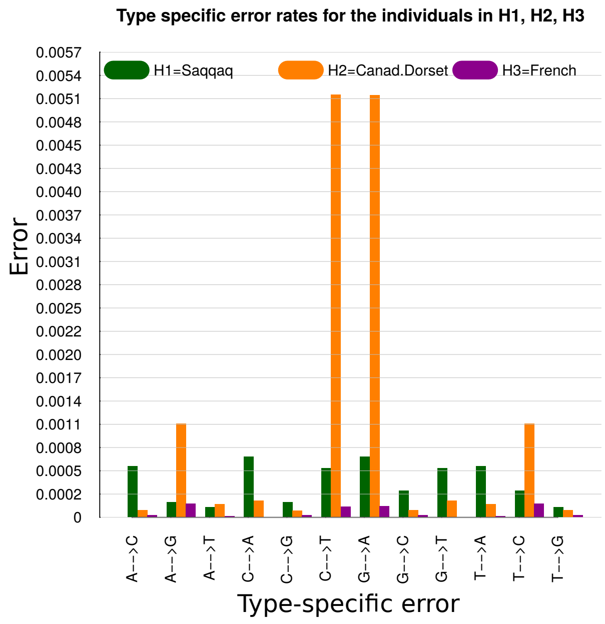

Figure S5. Behaviour of the D-Statistic in Function of the Type-Specific Error. Effect of increasing and decreasing the removal of error for the base transitions $C \rightarrow G$ and $C \rightarrow T$ for one of the Greenlandic Saqqaq, Canadian Dorset and French genomes. This corresponds to the addition of a value in the entry $\boldsymbol{e}(G, C)$ or $\boldsymbol{e}(T, C)$ of the estimated error matrix of one of the individuals, as if the estimated error rate was higher or lower. In solid lines are represented the values of D_{ext} for which the correction is performed. The dashed lines represent the analogous values where ancient transitions are not considered.

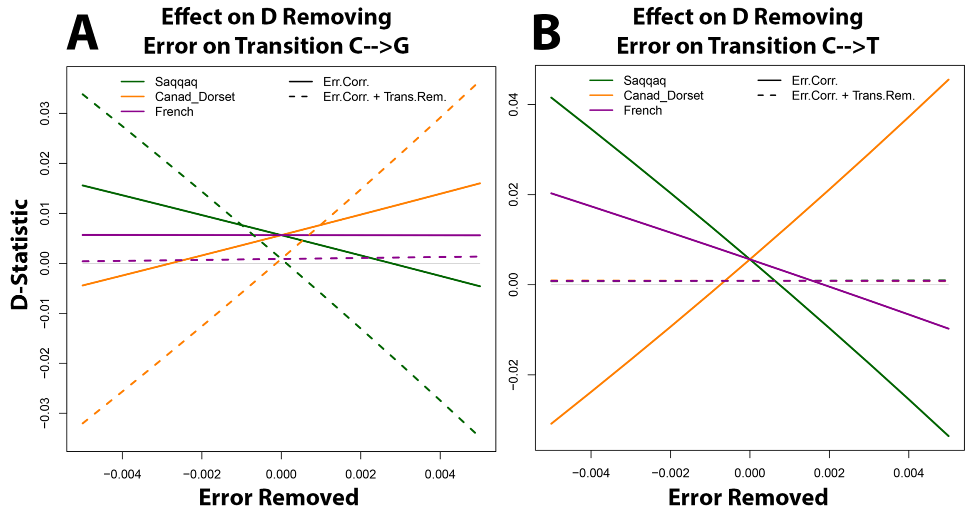

Supplement: Supplementary file 1 [file 551FileS1.pdf]
